# Efficacy of Praziquantel against *Schistosoma mekongi* and *Opisthorchis viverrini*: A Randomized, Single-Blinded Dose-Comparison Trial

**DOI:** 10.1371/journal.pntd.0001726

**Published:** 2012-07-24

**Authors:** Leonore Lovis, Tippi K. Mak, Khampheng Phongluxa, Phonepasong Ayé Soukhathammavong, Youthanavanh Vonghachack, Jennifer Keiser, Penelope Vounatsou, Marcel Tanner, Christoph Hatz, Jürg Utzinger, Peter Odermatt, Kongsap Akkhavong

**Affiliations:** 1 Laboratory of Parasitology, University of Neuchâtel, Neuchâtel, Switzerland; 2 Department of Epidemiology and Public Health, Swiss Tropical and Public Health Institute, Basel, Switzerland; 3 University of Basel, Basel, Switzerland; 4 National Institute of Public Health, Vientiane, Lao People’s Democratic Republic; 5 Parasitology Unit, Faculty of Basic Sciences, University of Health Sciences, Vientiane, Lao People’s Democratic Republic; 6 Department of Medical Parasitology and Infection Biology, Swiss Tropical and Public Health Institute, Basel, Switzerland; 7 Department of Medical Services and Diagnostic, Swiss Tropical and Public Health Institute, Basel, Switzerland; 8 Institute of Social and Preventive Medicine, University of Zurich, Zurich, Switzerland; Khon Kaen University, Thailand

## Abstract

**Background:**

Schistosomiasis and opisthorchiasis are of public health importance in Southeast Asia. Praziquantel (PZQ) is the drug of choice for morbidity control but few dose comparisons have been made.

**Methodology:**

Ninety-three schoolchildren were enrolled in an area of Lao PDR where *Schistosoma mekongi* and *Opisthorchis viverrini* coexist for a PZQ dose-comparison trial. Prevalence and intensity of infections were determined by a rigorous diagnostic effort (3 stool specimens, each examined with triplicate Kato-Katz) before and 28–30 days after treatment. Ninety children with full baseline data were randomized to receive PZQ: the 40 mg/kg standard single dose (*n* = 45) or a 75 mg/kg total dose (50 mg/kg+25 mg/kg, 4 hours apart; *n* = 45). Adverse events were assessed at 3 and 24 hours posttreatment.

**Principal Findings:**

Baseline infection prevalence of *S. mekongi* and *O. viverrini* were 87.8% and 98.9%, respectively. *S. mekongi* cure rates were 75.0% (95% confidence interval (CI): 56.6–88.5%) and 80.8% (95% CI: 60.6–93.4%) for 40 mg/kg and 75 mg/kg PZQ, respectively (*P* = 0.60). *O. viverrini* cure rates were significantly different at 71.4% (95% CI: 53.4–84.4%) and 96.6% (95% CI: not defined), respectively (*P* = 0.009). Egg reduction rates (ERRs) against *O. viverrini* were very high for both doses (>99%), but slightly lower for *S. mekongi* at 40 mg/kg (96.4% *vs.* 98.1%) and not influenced by increasing diagnostic effort. *O. viverrini* cure rates would have been overestimated and no statistical difference between doses found if efficacy was based on a minimum sampling effort (single Kato-Katz before and after treatment). Adverse events were common (96%), mainly mild with no significant differences between the two treatment groups.

**Conclusions/Significance:**

Cure rate from the 75 mg/kg PZQ dose was more efficacious than 40 mg/kg against *O. viverrini* but not against *S. mekongi* infections, while ERRs were similar for both doses.

**Trial Registration:**

Controlled-Trials.com ISRCTN57714676

## Author Summary

Parasitic worm infections are of public health importance in Southeast Asia. Particularly, the blood-dwelling *Schistosoma mekongi* worm, which is acquired by skin contact with the infectious cercariae in freshwater, can lead to liver enlargement. An infection with *Opisthorchis viverrini* is obtained by consumption of undercooked freshwater fish, and this infection increases the risk of developing cholangiocarcinoma. A single oral dose of 40 mg/kg praziquantel is recommended for mass treatment of schistosomiasis and opisthorchiasis, while at the individual level, a total dose of 75 mg/kg divided into three doses, is currently common practice to treat *O. viverrini* infection. Diagnosis is based on stool examination under a microscope for detection of worm eggs, but is limited by the low sensitivity of the widely used Kato-Katz technique. In this study, we showed that a 75 mg/kg total dose of praziquantel (50 mg/kg+25 mg/kg given 4 hours apart) cleared significantly more *O. viverrini* infections than a single 40 mg/kg dose, but no difference was observed for *S. mekongi*. Solicited adverse event profiles were mainly mild and similar in both groups. Repeated stool examination before and after treatment was essential for an accurate assessment of drug efficacy in terms of cure rate, but showed no effect on assessing egg reduction rates.

## Introduction

Schistosomiasis, food-borne trematodiasis, and soil-transmitted helminthiasis are neglected tropical diseases that are of considerable public health relevance in Southeast Asia [1]. In Lao People's Democratic Republic (Lao PDR), approximately 80,000 individuals are at risk for schistosomiasis mekongi, 2 million individuals are at risk for food-borne trematodiasis (particularly opisthorchiasis), and 1 million school-aged children are at risk for soil-transmitted helminthiasis [1]. Praziquantel (PZQ) is the current drug of choice in the treatment of schistosomiasis and most of the food-borne trematode infections [1]. Deworming programs against schistosomiasis aim at morbidity control [2]. The World Health Organization (WHO) recommends a standard single dose of oral PZQ between 40 and 60 mg/kg for both schistosomiasis and food-borne trematodiasis [1], [2]. In Lao PDR, a single dose of 40 mg/kg PZQ is recommended for mass treatment of schistosomiasis and opisthorchiasis [3]. For individual treatment, the PZQ dose to treat *Opisthorchis viverrini* infection is a total dose of 75 mg/kg divided into three doses [4].

PZQ is known to be effective against all six *Schistosoma* species causing disease in humans. However there have been just two small published clinical trials on PZQ cure rates against *Schistosoma mekongi* [5], [6]. Both were non-randomized studies involving individuals relocated to non-endemic areas and given 60 mg/kg PZQ divided into two or three doses. To our knowledge, a controlled trial to treat *S. mekongi* using 40 mg/kg, the recommended dose for mass treatment in Lao PDR, and any comparison between different PZQ doses for superiority has so far not been undertaken.

Several clinical trials have assessed PZQ efficacy against *O. viverrini* at the following dosages: single dose of 25, 40, or 50 mg/kg, or repeated 25 mg/kg doses for a total dose of 50, 75, or 150 mg/kg [7]–[13]. However, none has been conducted in Lao PDR, which also has *S. mekongi* co-endemic areas, and 40 mg/kg has not been compared with 75 mg/kg.

Diagnosis of schistosomiasis, opisthorchiasis, and other intestinal or hepatobiliar helminth infections in epidemiological studies is commonly based on the detection of parasite eggs in stool specimens under a microscope. The Kato-Katz technique [14], [15] is the recommended field method [16] and permits estimation of infection intensity expressed in eggs per gram of feces (EPG). It is a relatively simple and rapid diagnostic method, but unfortunately, a single Kato-Katz thick smear has low sensitivity, particularly for light infections, and hence repeated stool examinations are necessary to improve the sensitivity of this technique [17]–[20]. This is especially important after treatment to avoid overestimation of cure rates. The low sensitivity of a single Kato-Katz thick smear results from the small amount of stool examined (usually 41.7 mg), variation in helminth egg excretion over time in the same individual, and from variation in egg density within a stool specimen depending on sampling location, as recognized for *Schistosoma mansoni* [19], [21], [22]. The relative contribution of day-to-day and intra-specimen variation in fecal egg counts has been investigated for *S. mansoni* [19], [21] where examination of repeated stool specimens, rather than examination of multiple Kato-Katz thick smears derived from a single stool specimen, was shown to be more appropriate to improve the sensitivity of detecting an infection [19], [22]. While it is documented for *S. mansoni* that diagnostic sensitivity depends on the sampling effort, other helminth species are less well investigated. Repeated or multiple stool specimen collection is difficult in practice, particularly in rural community field surveys [20], due to logistical requirements and cost implications.

The current study pursued two objectives. First, we assessed the efficacy of two oral PZQ regimens (i.e., 40 mg/kg single dose, and 75 mg/kg divided dose, given as 50 mg/kg then 25 mg/kg 4 hours apart) against *S. mekongi* and *O. viverrini* infections. Second, we determined the effect of multiple stool sampling on the diagnostic accuracy of the Kato-Katz technique before and after treatment, and assessed its impact on drug efficacy evaluation, considering both cure and egg reduction rates.

## Methods

### Ethics Statement

Ethical clearance was obtained from the National Ethics Committee, Ministry of Health (MoH) in Vientiane, Lao PDR (reference no. 027/NECHR) and by the Ethics Committee of Basel, Switzerland (EKBB; reference no. 255/06). The study protocol is registered with Current Controlled Trials on controlled-trials.com (identifier ISRCTN57714676). Written informed consent was obtained by the parents or guardians of all pupils before participation in the study. The children had the opportunity to withdraw from the study at any time.

Both doses of PZQ (i.e., single 40 mg/kg dose or total of 75 mg/kg dose) are accepted within Lao MoH published guidelines. The 40 mg/kg single dose is mainly used in mass drug administration programs, while 75 mg/kg (divided into three dosages) is used for the treatment of individuals. In our study the 75 mg/kg dose was divided into two doses (50 mg/kg plus 25 mg/kg given 4 hours apart) to simplify the regimen for a school setting where classes ended by the early afternoon. At the end of the follow-up period, all children were treated against soil-transmitted helminth infections with a single oral dose of 400 mg albendazole [3].

### Study Outcomes

The primary objective of this study was to compare the efficacy of two different dose regimens of oral PZQ in school-aged children from southern Lao PDR in a *S. mekongi* and *O. viverrini* co-endemic area. The two regimens compared were (i) 40 mg/kg single dose and (ii) 75 mg/kg divided dose, given as 50 mg/kg then 25 mg/kg 4 hours apart. The secondary objectives were to determine the effect of multiple stool sampling to assess cure and egg reduction rates and to estimate the increased diagnostic sensitivity by multiple Kato-Katz thick smears from a single stool specimen compared with additional stool specimens obtained over several days before and after treatment. *S. mekongi* and *O. viverrini* were the species of primary interest, but hookworm was also included for the baseline analyses. Finally, the prevalence of the other intestinal helminth infections among our cohort of schoolchildren was also assessed.

### Study Design, Sample Size Calculation, and Population

The dose comparison study was a randomized trial with 1∶1 allocation. It was conducted in February and March 2007 in the primary and secondary schools on Don Long Island, Khong district, Champasack province, Lao PDR. The 308 children registered at the Don Long school were invited for the dose comparison trial. Most of the pupils (60%) lived in one of the four villages of Don Long Island, whereas the remaining children traveled from four villages on surrounding islands. In-depth stool examination was limited to 93 children aged 10–15 years (two classes). Based on the asymptotic normal method (formula 7) of Sahai and Khurshid [23], this sample size has a 70% power to demonstrate a superiority of 20% of the highest PZQ dosage (type I error: alpha = 5%; 1-tailed test) when considering a 20% dropout rate. Analyses of the present paper are restricted to this in-depth cohort. Acutely ill or febrile children were excluded from the study.

Don Long is a rural island in the Mekong River with about 1,500 inhabitants who practice subsistence farming and fishing. Previous studies on this island found the area to be co-endemic for *S. mekongi* and *O. viverrini* infections [24], [25]. Laboratory facilities were established in Khong district hospital in Muang Khong, a village on the east side of Don Khong, the main island of Khong district.

### Treatment: Allocation, Randomization, Dose Preparation, and Blinding

The children were assigned into two treatment arms following a 1∶1 allocation regardless of the baseline examination. Randomization was generated using a random number table in blocks of 10. Randomization and supervision of the trial were conducted by the study leaders (LL, TKM). Based on the child's weight, the dose was rounded to the nearest 150 mg by splitting the 600 mg PZQ tablets (Distocide®, Korea) in quarters using a pill cutter. Doses were prepared in advance by team members not involved in administrating the intervention. Each preparation was double verified for name, dose, and recorded weight for each child. Twelve hours before treatment, all doses were prepared and sealed in opaque envelopes that were labeled with the dose number, study unique identification number, the child's name, and weight. After the dose envelopes were prepared, the randomization and allocation list was sealed in an opaque envelope. Box 1 contained the envelopes with the first (and only) dose for children allocated in the 40 mg/kg arm and the first dose for those assigned to the 75 mg/kg arm, organized by school class and name. Box 2 contained the prepared envelopes for the second dose (25 mg/kg) only for those children allocated for the total dose of 75 mg/kg PZQ.

The drugs were administered by one of two paired teams of health care workers. The team confirmed that the child matched the identification on the drug envelope and then directly observed treatment. The drug administering teams were not involved prior to or after the study and not in any outcome assessments. As the different regimen was apparent (single *vs.* a divided dose 4 hours apart) neither the two health care teams nor the children were masked during treatment administration. The Lao physicians who assessed the children for adverse events following treatment were unaware of the dose allocation and were not involved with administering the intervention (KP, PAS). Laboratory technicians assessing infection status were blinded to the dose allocation.

### Study Procedures

The purpose and procedures of the study were explained to the school director, teachers, and to the village chief, who all agreed to participate. The study was explained during class to the children and written informed consent was received from their parents or guardians.

Clinical baseline measurements and baseline laboratory determination of infection status were performed prior to treatment for each participating child. Clinical measurements included a morbidity questionnaire and physical examination. For laboratory procedures, plastic bags with pre-labeled 30 ml plastic containers were distributed to the children at enrolment and pupils were asked to return the containers the following day with a thumb-sized portion of their morning stools. Containers were collected each morning at the school from 07:30 to 08:30 hours, recorded on a line listing, and children were given new empty plastic containers for the following day. This procedure was repeated until 3 morning stool specimens per child were received. Fresh stool specimens were transferred daily to the laboratory on Khong Island for examination. From each stool specimens, triplicate Kato-Katz thick smears using standard 41.7 mg templates were prepared on microscope slides in accordance with the kit instructions (Vestergaard Frandsen; Lausanne, Switzerland). The slides were quantitatively examined under a microscope within 1 hour following slide preparation. The number of eggs of *O. viverrini*, *S. mekongi*, hookworm, *Trichuris trichiura*, *Ascaris lumbricoides*, *Taenia* spp., *Enterobius vermiculari*s, and other helminths were counted and recorded separately. For quality control, 10% of the slides were randomly selected and re-examined by a senior technician without prior knowledge of the results. When discrepancies were observed (e.g., egg counts differing by more than 10%), the technicians received closer supervision by a more experienced colleague. Since *O. viverrini* cannot be easily distinguished from minute intestinal flukes (MIF) microscopically by the Kato-Katz technique [26], infections reported here as *O. viverrini* infections are assumed to include some MIF co-infections.

Following baseline data collection, children were treated with 40 mg/kg or 75 mg/kg oral PZQ as described. Immediately following the dose, the children were given two soupspoons of sticky rice (∼40 g) to increase PZQ bioavailability and minimize potential adverse events [27]. Adverse events spontaneously reported within 3 hours after administration of the first dose were recorded. Additionally, a solicited questionnaire on adverse events was administered 24 hours following PZQ administration and graded for severity. All clinical and laboratory assessments were repeated 28–30 days after PZQ administration.

### Statistical Analysis

Data were entered in EpiData software version 3.1 (EpiData Association; Odense, Denmark) and double-checked against the original data sheets. Data analysis was performed using Intercooled STATA release 9.0 (StataCorp; College Station, TX, USA).

For each helminth species, an infection was defined as the presence of one or more eggs in at least one of the Kato-Katz thick smears examined. Cumulative prevalence of each helminth infection detected after examination of 9 Kato-Katz thick smears (3 stool specimens with triplicate Kato-Katz per specimen) was calculated. Tests for significant associations with gender were analyzed by negative binomial regression. Intensity of infection (expressed in EPG) was calculated by multiplying the observed number of eggs by a factor of 24. Geometric mean intensity of infection was calculated on EPG. Infections with *O. viverrini* were classified into three groups [28]: light (1–999 EPG), moderate (1,000–9,999 EPG), and heavy infections (≥10,000 EPG). *S. mekongi* infections were grouped into the following three categories [29]: light (1–99 EPG), moderate (100–399 EPG), and heavy infections (≥400 EPG). Negative binomial regression was applied to compare infection intensities of *S. mekongi* and *O. viverrini* at baseline among the two treatment groups.

Cure rates of *S. mekongi* and *O. viverrini* were calculated as the proportion of children with no egg excretion after treatment among those with eggs in their stool at baseline. Children found egg-negative prior to treatment but egg-positive after treatment were considered to be false negative and counted as infected at baseline. These infections were assumed to have been missed at baseline because the 28–30 days follow-up would not have provided adequate time for re-infection and patency between the two surveys. Cure rates obtained with the two tested doses were compared with Fisher's exact test. Egg reduction rates were determined by comparing the geometric mean egg output before and 28–30 days after treatment among children infected at baseline (1 - geometric mean egg output posttreatment/geometric mean egg output at baseline, multiplied by 100).

The effect of multiple sampling on the sensitivity of the Kato-Katz technique to detect *S. mekongi* and *O. viverrini* infections was assessed before and after drug administration. Hookworm infections were also included at baseline. Prevalences with 95% confidence interval (CI) were calculated for each sampling effort, the minimum effort being defined as the first Kato-Katz thick smear derived from the first stool specimen. The sampling effort increased with additional Kato-Katz thick smear examinations from the same stool specimen and with additional stool specimens. The McNemar test was used to compare prevalences assessed by different sampling efforts. The maximum sampling effort, 9 Kato-Katz thick smears, was taken as the diagnostic ‘gold’ standard to assess the sensitivity of increasing sampling efforts.

Adverse event frequencies depending on treatment doses were compared with the exact χ^2^ test. Additionally, infection intensities were expressed in EPG and for each child the arithmetic means were computed for each sampling effort. At the cohort level, geometric mean fecal egg counts were calculated for each sampling effort considering only the children with complete datasets at each time point separately. The analysis was restricted to the egg-positive children, based on the examination of 9 Kato-Katz thick smears (maximum sampling effort).

## Results

**Figure 1:**
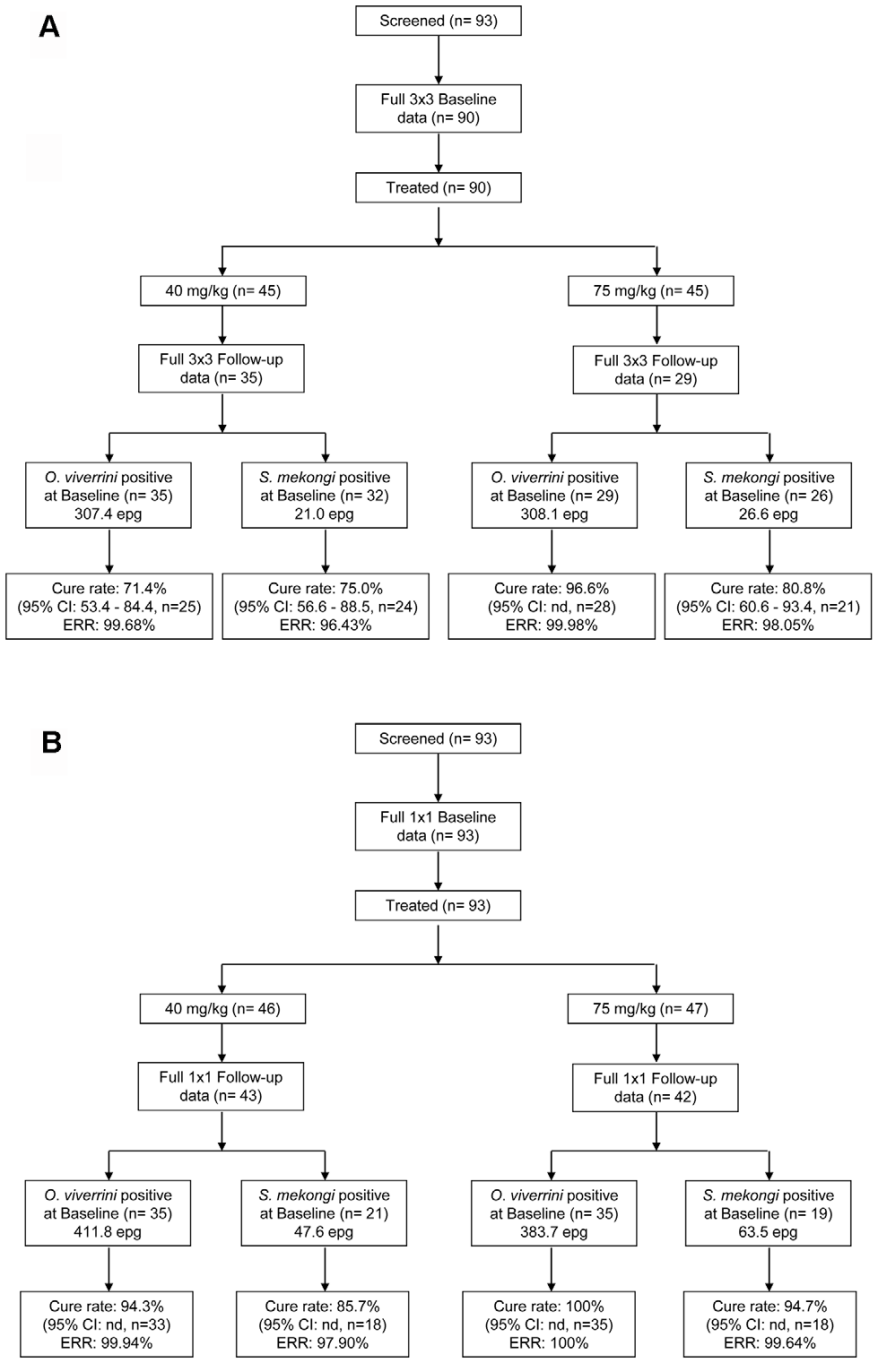
Flowchart of subjects with cure and egg reduction rates. Cure and egg reduction rates are presented for *O. viverrini* and *S. mekongi* infections following 40 mg/kg and 75 mg/kg (50 mg/kg+25 mg/kg 4 hours apart) PZQ treatment considering (a) the maximum sampling effort (3×3, 3 stool specimens with triplicate Kato-Katz thick smears per specimen); (b) the minimum sampling effort (1×1, single Kato-Katz thick smear from the first stool specimen).

The 93 children (54 boys, 39 girls) included in the in-depth cohort all agreed to participate and written parental or guardian consent was received. Participants had a median age of 12 years (range: 10–15 years). Eighty-five children provided at least one stool specimen during the baseline survey and during the 28–30 day posttreatment follow-up. Among them, 64 children provided three stool specimens at both time points and had therefore complete datasets, with a compliance of 69% (64/93) (see Figure 1).

All schoolchildren were given treatment, according to their randomized treatment allocation. In the in-depth cohort, 46 children received 40 mg/kg PZQ and 47 received 75 mg/kg divided dose. The effect of multiple sampling on the sensitivity of the Kato-Katz technique was analyzed before and after treatment and was restricted to children with complete datasets at each time point separately, with a compliance of 97% (90/93) at baseline and 71% (66/93) at the 28–30 day posttreatment follow-up. There were no significant differences in the gender ratio, average age, or infection prevalence between the baseline and the posttreatment follow-up groups (all *P*>0.05).

### Helminth Infection at Baseline

Table 1 summarizes baseline infection prevalences and intensities of all helminth species diagnosed in the present study before PZQ administration. Results pertained to those children who had complete data records (9 Kato-Katz thick smears) prior to treatment (*n* = 90) and before and after treatment combined (*n* = 64). *S. mekongi*, *O. viverrini*, and hookworm were the most common parasitic infections at baseline, with prevalences above 85% for each helminth species, as assessed with the maximum sampling effort. Other intestinal parasitic infections, in descending order of prevalence, were *T. trichiura*, *A. lumbricoides*, *E. vermicularis*, and *Taenia* spp. One infection with *Hymenolepis diminuta* was detected. Infection prevalences for any of the aforementioned helminths did not differ between boys and girls.

**Table 1:** Baseline prevalence of infection of the main helminth species and infection intensity among egg-positive children.

	Full 3×3 data at baseline (*n* = 90)				Full 3×3 data at baseline and 28–30 days posttreatment follow-up (*n* = 64)			
Helminth species	Prevalence (%)	95% CI	Infection intensity (EPG)	95% CI	Prevalence (%)	95% CI	Infection intensity (EPG)	95% CI
*S. mekongi*	87.8	79.2–93.7	25	18–33	85.9	75.0–93.4	28	20–40
*O. viverrini*	98.9	n.d.	342	229–510	98.4	n.d.	337	201–566
Hookworm	96.7	n.d.	321	221–464	95.3	n.d.	252	157–403
*T. trichiura*	23.3	15.1–33.4	13	7–24	18.8	10.1–30.5	9	4–21
*A. lumbricoides*	7.8	3.2–15.4	124	9–1,506	6.3	n.d.	16	1–141
*E. vermicularis*	7.8	3.2–15.4	12	2–53	6.3	n.d.	10	0–242
*Taenia* spp.	6.7	2.5–13.9	6	2–17	4.7	n.d.	9	0–112

Study was carried out among 93 children in primary and secondary schools on Don Long Island, Khong district, Champasack province, Lao PDR in February and March 2007. Full 3×3 data refers to children who provided 3 stool specimens over consecutive days, with triplicate Kato-Katz thick smear examinations per stool specimen.

CI, confidence interval; EPG, eggs per gram of stool; n.d., not defined.

### Praziquantel Cure and Egg Reduction Rates against *S. mekongi* and *O. viverrini*

Cure and egg reduction rates were compared between two cohorts (Figures 1a and 1b). First, children who complied with the maximum diagnostic effort (9 Kato-Katz thick smears before and after treatment, *n* = 64) and, second, children with a minimum diagnostic effort (1 Kato-Katz thick smear at each time point, *n* = 85). Results are summarized in Tables 2 and 3. For both cohorts, there was no significant differences in the infection intensities of *S. mekongi* and *O. viverrini* at baseline between the two treatment groups (all *P*>0.05).

**Table 2:** *S. mekongi* infection intensity before (D0) and posttreatment (D28) and egg reduction rate for maximum and minimum diagnostic effort.

Treatment		Maximum diagnostic effort (3×3 datasets, *n* = 64)						Minimum diagnostic effort (1×1 datasets, *n* = 85)					
	Infection intensity	n	# cured	% cured	D0 GM (EPG)	D28 GM (EPG)	ERR (%)	n	# cured	% cured	D0 GM (EPG)	D28 GM (EPG)	ERR (%)
40 mg/kg	All infections	32	24	75.0	21.0	0.75	96.4	21	18	85.7	47.6	1.00	97.9
	1–99 EPG	27	20	74.1	13.1	0.81	93.8	16	13	81.3	22.4	1.48	93.4
	100–399 EPG	3	3	100	157.1	0	100	2	2	100	200.8	0	100
	≥400 EPG	2	1	50.0	451.3	1.52	99.7	3	3	100	923.0	0	100
75 mg/kg	All infections	26	21	80.8	26.6	0.52	98.1	19	18	94.7	63.5	0.23	99.6
	1–99 EPG	22	17	77.3	18.2	0.64	96.5	14	13	92.9	45.1	0.32	99.3
	100–399 EPG	4	4	100	201.7	0	100	5	5	100	163.6	0	100
	≥400 EPG	0	0	na	na	na	na	0	0	na	na	na	na

EPG, eggs per gram of stool; ERR, egg reduction rate; GM, geometric mean; na, not applicable.

**Table 3:** *O. viverrini* infection intensity before (D0) and posttreatment (D28) and egg reduction rate for maximum and minimum diagnostic effort.

Treatment		Maximum diagnostic effort (3×3 datasets, *n* = 64)						Minimum diagnostic effort (1×1 datasets, *n* = 85)					
	Infection intensity	n	# cured	% cured	D0 GM (EPG)	D28 GM (EPG)	ERR (%)	n	# cured	% cured	D0 GM (EPG)	D28 GM (EPG)	ERR (%)
40 mg/kg	All infections	35	25	71.4	307.4	0.99	99.7	35	33	94.3	411.8	0.26	99.94
	1–999 EPG	24	18	75.0	96.4	0.48	99.5	24	24	100	142.3	0	100
	1,000–9,999 EPG	9	6	66.7	2,460	2.1	99.92	9	7	77.8	2,817	1.4	99.95
	≥10,000 EPG	2	1	50.0	27,344	9.0	99.97	2	2	100	23,778	0	100
75 mg/kg	All infections	29	28	96.6	308.1	0.05	99.98	35	35	100	383.7	0	100
	1–999 EPG	20	20	100	103.7	0.00	100	24	24	100	181.1	0	100
	1,000–9,999 EPG	9	8	88.9	3,424.2	0.16	100	10	10	100	1,568	0	100
	≥10,000 EPG	0	0	na	na	na	na	1	1	100	18,816	0	100

EPG, eggs per gram of stool; ERR, egg reduction rate; GM, geometric mean; na, not applicable.

*S. mekongi* cure rates among children who had provided three stool specimens at baseline and follow-up were 80.8% (21/26; 95% CI: 60.6–93.4%) after 75 mg/kg PZQ and 75.0% (24/32; 95% CI: 56.6–88.5%) after 40 mg/kg PZQ, which was not significantly different (*P* = 0.754). With the minimum diagnostic effort, observed cure rates were considerably higher, 94.7% (18/19; 95% CI: not defined) and 85.7% (18/21; 95% CI: not defined), respectively. *S. mekongi* egg reduction rates in both cohorts were >93%. Slightly higher egg reduction rates were observed at the minimum sampling effort (97.9% and 99.6% in the 40 mg/kg and 75 mg/kg treatment group, respectively), compared to the highest sampling effort (96.4% and 98.1%, respectively).

Based on the maximum sampling effort, *O. viverrini* cure rates were 96.6% (28/29; 95% CI: not defined) after 75 mg/kg PZQ and 71.4% (25/35; 95% CI: 53.4–84.4%) after 40 mg/kg PZQ, showing a statistically significant difference (*P* = 0.009). Considering the minimum diagnostic effort, observed cure rates were 100% (35/35; 95% CI: not defined) and 94.3% (33/35; 95% CI: not defined), respectively, with no statistically significant difference (*P* = 0.493). Egg reduction rates, regardless of treatment group and diagnostic efforts, were above 99%.

### Adverse Events

Solicited 24-hour adverse event profiles in the two treatment groups are summarized in Table 4. Fourteen children were not available to be interviewed (*n* = 6, 40 mg/kg dose; *n* = 8, 75 mg/kg dose), corresponding to 15.1% lost to follow-up, but no serious adverse events were reported by the community when we returned days 28–30 for post-treatment follow-up. Most children reported one or more adverse events (76/79, 96%). More cases were reported for most types of adverse events in the 75 mg/kg treatment arm, but did not reach statistical significance in this small sample when comparing the total number of events or those graded as severe. There were a total of 7 cases recorded as hypotension (below 100 mm Hg systolic blood pressure) in the 75 mg/kg treatment group compared with a single case in the 40 mg/kg group, which was statistically higher (*P*<0.02) but no case was graded severe (e.g., no syncope). Children with hypotension associated with dizziness and vomiting were given rest and monitored; all cases were self-limiting. No serious adverse events required hospitalization.

**Table 4:** Solicited adverse events reported 24 hours following PZQ administration (*n* = 93).

		Total no. of reports			Reports graded as severe		
Organ class	Symptoms	40 mg/kg	75 mg/kg	*p* *	40 mg/kg	75 mg/kg	*p* *
		(*n* = 40)	(n = 39)		(*n* = 40)	(*n* = 39)	
Systemic	Allergic reaction	1	0	0.32	0	0	
	Fever	0	2	0.15	0	0	
	Headache	27	31	0.23	0	0	
	Anxiety	1	2	0.54	0	0	
	Fatigue	24	28	0.27	0	0	
	Vertigo/dizziness	20	23	0.42	0	1	0.31
Gastro-intestinal	Nausea	9	12	0.41	0	0	
	Vomiting	5	11	0.08	0	2	0.15
	Diarrhea	7	5	0.56	0	0	
	Constipation	2	0	0.16	0	0	
	Abdominal pain	23	27	0.28	0	0	
Cardiovascular	Palpitations	4	6	0.47	0	0	
	Hypotension	1	7	0.02	0	0	
Respiratory	Cough	1	1	0.99	0	0	
	Bronchospasm	1	1	0.99	0	0	
	Dyspnea	1	1	0.99	0	0	

*: according to exact χ^2^ test.

The two study groups were 40 mg/kg *vs.* 75 mg/kg divided into 2 doses of 50 mg/kg+25 mg/kg, 4 hours apart.

### Effect of Multiple Sampling Efforts on Cumulative Prevalence

**Figure 2:**
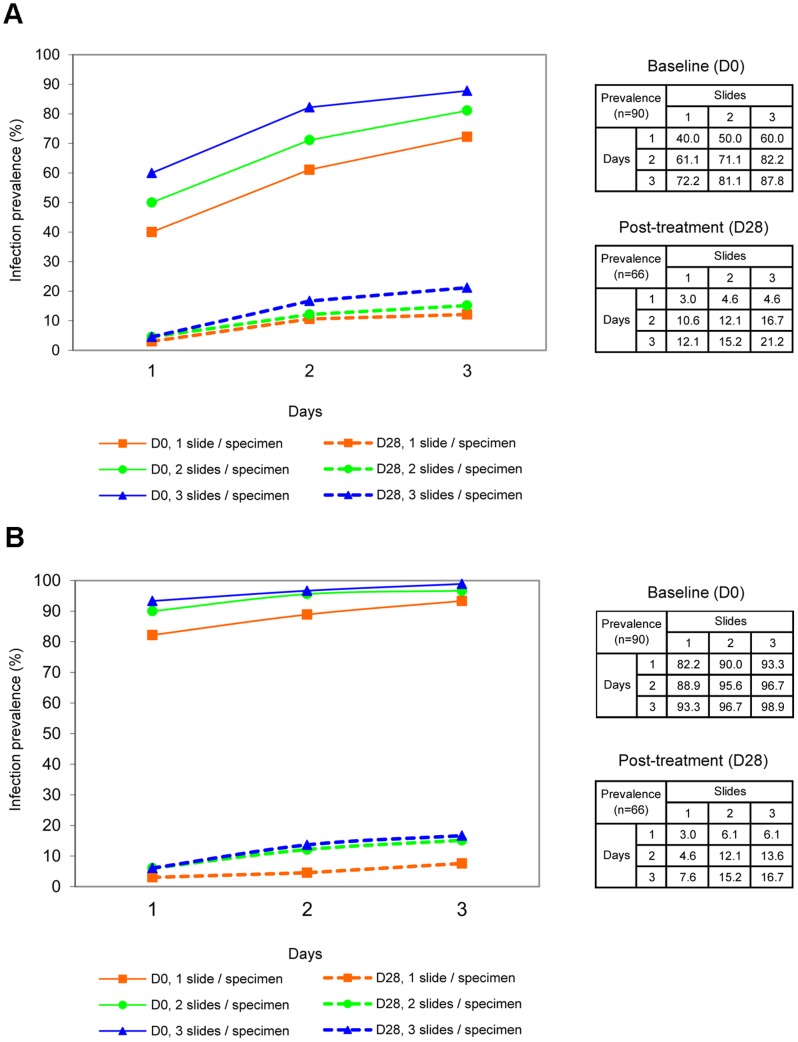
Cumulative prevalence according to the sampling effort. Cumulative infection prevalences for (a) *S. mekongi* and (b) *O. viverrini* by the number over consecutive days of stool specimen collection (x-axis). Each point on a curve represents a cumulative prevalence value for each sampling effort (number of Kato-Katz thick smears per stool specimen). At baseline (day 0), *n* = 90; after treatment (days 28–30), *n* = 66.

Figure 2 shows the cumulative prevalence of infected children over repeated stool specimens according to the number of Kato-Katz thick smears examined per stool specimen for *S. mekongi* and *O. viverrini* infections both at baseline and at the 28–30 day posttreatment follow-up survey. Baseline results for hookworm infections were also recorded although not the primary outcome of the study (nor were hypotheses made on the efficacy of PZQ against this helminth species). The sensitivity of three different sampling efforts (considering the maximum diagnostic effort of 9 Kato-Katz thick smears as the diagnostic ‘gold’ standard) is presented in Table 5.

**Table 5:** Sensitivity of different sampling efforts to detect *S. mekongi* and *O. viverrini* infections.

	Sensitivity of different Kato-Katz thick smear sampling efforts							
Helminth species	Baseline survey (*n* = 90)				Days 28–30 posttreatment follow-up (*n* = 66)			
	1 stool1 smear	1 stool3 smears	3 stools1 smear	3 stools3 smears	1 stool1 smear	1 stool3 smears	3 stools1 smear	3 stools3 smears
	n (%)	n (%)	n (%)	n (‘gold’ standard)	n (%)	n (%)	n (%)	n (‘gold’ standard)
*S. mekongi*	36 (45.6)	54 (68.4)	65 (82.3)	79 (100)	2 (14.3)	3 (21.4)	8 (57.1)	14 (100)
*O. viverrini*	74 (83.1)	84 (94.4)	84 (94.4)	89 (100)	2 (18.2)	4 (36.4)	5 (45.4)	11 (100)

Study was carried out among 93 children in primary and secondary schools on Don Long Island, Khong district, Champasack province, Lao PDR in February and March 2007. Sensitivity is compared before (*n* = 90) and after PZQ administration (*n* = 66), using the maximum sampling effort as the diagnostic ‘gold’ standard for the following sampling efforts: 1×1 sampling effort examines the first Kato-Katz thick smear only; 1×3 examines the first stool specimen by triplicate Kato-Katz thick smears; 3×1 examines 3 stool specimens by a single Kato-Katz thick smear for each specimen.

#### At baseline

Baseline prevalence of *S. mekongi* infection increased more than two-fold when assessed with the maximal sampling effort (87.8%; 95% CI: 79.2–93.7%) compared with the minimal sampling effort (40.0%; 95% CI: 29.8–50.9%), suggesting a sensitivity of the first Kato-Katz thick smear of 45.6% (95% CI: 34.3–57.2%). By contrast, baseline prevalences of *O. viverrini* and hookworm infections assessed with the minimum sampling effort were already very high (82.2%, 95% CI: 72.7–89.5%; and 81.1%, 95% CI: 71.5–88.6%, respectively), and reached 98.9% (95% CI: not defined) and 96.7% (95% CI: not defined) when assessed with the maximum sampling effort. Hence, corresponding sensitivities of the first Kato-Katz thick smear were 83.1% (95% CI: 73.7–90.2%) and 83.9% (95% CI: 74.5–90.9%), respectively. For all three helminth species, examination of triplicate Kato-Katz thick smears from the first stool specimen (1×3 sampling scheme) or examination of one Kato-Katz thick smear per stool specimen over three specimens (3×1) led to substantial increases in the cumulative prevalence estimate in comparison with a single Kato-Katz thick smear (*P*<0.01).

The baseline prevalence for *S. mekongi* infection, as assessed by three stool specimens , each subjected to a single Kato-Katz thick smear (3×1), was 72.2% (95% CI: 61.8–81.1%). This was significantly higher than a single stool specimen examined by triplicate Kato-Katz thick smears (1×3) revealing a prevalence of 60.0% (95% CI: 49.1–70.2%; *P* = 0.028).

For hookworm detection, the prevalence slightly increased from 88.9% (95% CI: 80.5–94.5%) to 94.4% (95% CI: 87.5–98.2%, P = 0.059). No difference was found for *O. viverrini* infection prevalence comparing the two different sampling schemes (93.3%; 95% CI: 86.1–97.5% in both cases).

#### 28–30 days after treatment

**Figure 3:**
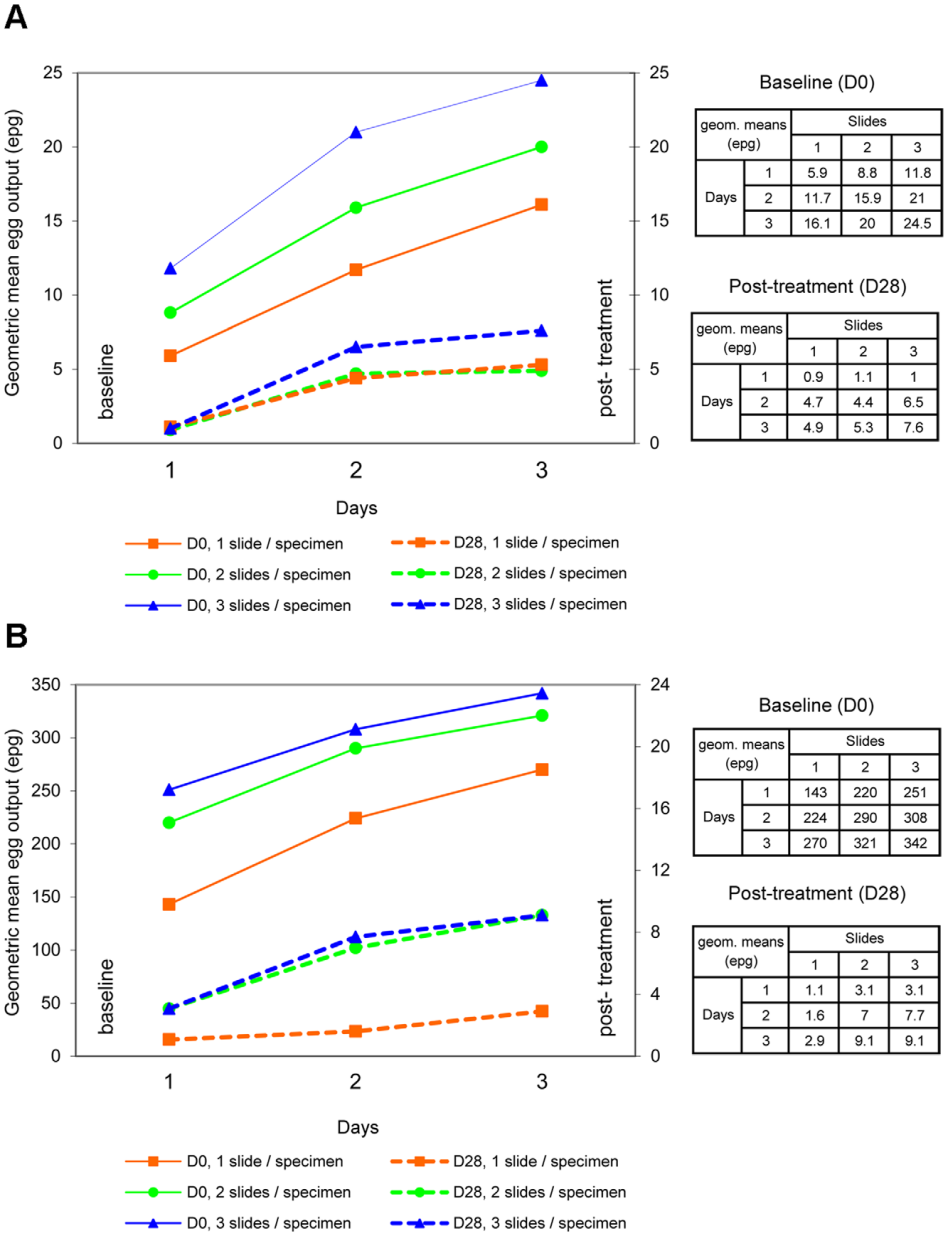
Geometric mean fecal egg counts according to the sampling effort. Geometric mean fecal egg counts before and after PZQ treatment, by the number of days of stool specimen collection (x-axis), based on children diagnosed “infected” following maximum Kato-Katz thick smear sampling effort. (a) *S. mekongi* infected at baseline (day 0), *n* = 79; days 28–30 after treatment, *n* = 14; and (b) *O. viverrini* infected at baseline (day 0), *n* = 89; days 28–30 after treatment, *n* = 11. Each point on a curve represents the geometric mean fecal egg count for each sampling effort (number of Kato-Katz thick smears examined per stool specimen).

After PZQ treatment, the effect of stool sampling effort showed a stronger relative increase in detecting helminth infections than at the pretreatment baseline survey. Figures 2 and 3 show that the *S. mekongi* infection prevalence rose seven-fold from 3.0% (95% CI: not defined) to 21.2% (95% CI: 12.1–33.0%), and the *O. viverrini* infection prevalence showed over a five-fold increase from 3.0% (95% CI: not defined) to 16.7% (95% CI: 8.6–27.9%), when comparing results from minimum and maximum sampling efforts. The sensitivity of a single Kato-Katz thick smear (1×1) was only 14.3% (95% CI: not defined) and 18.2% (95% CI: not defined) for *S. mekongi* and *O. viverrini*, respectively (Table 5). Similar to baseline, a 3×1 sampling effort led to a moderately higher sensitivity than a 1×3 sampling effort (*P* = 0.059) to detect *S. mekongi* eggs, but there was no significant difference in sensitivity to detect *O. viverrini* eggs.

### Effect of Multiple Sampling Efforts on Mean Fecal Egg Counts

Figure 3 illustrates the results of increased sampling effort on the geometric mean fecal egg counts before and after PZQ treatment of all children infected with *S. mekongi* and *O. viverrini*. This was also assessed for hookworm at baseline.

At baseline, the mean fecal egg counts gradually increased with increasing sampling efforts. Thus, egg count estimates for *S. mekongi, O. viverrini*, and hookworm increased 4, 2.4 and 1.7-fold, reaching values of 25 EPG, 342 EPG and 321 EPG, respectively, when assessed with the maximum sampling effort. *S. mekongi* and *O. viverrini* mean fecal egg count estimates were considered low-intensity infections.

After PZQ treatment, the benefit of the maximum sampling effort for EPG was 9-fold and 8-fold for *S. mekongi* and *O. viverrini*, respectively. When comparing the pretreatment baseline with the 28–30 day posttreatment follow-up, the mean fecal egg count for *O. viverrini* sharply decreased from 342 to 9.1 EPG. The decrease was less marked for *S. mekongi*, from 25 to 8 EPG.

A 3×1 sampling effort yielded substantially higher estimates than a 1×3 sampling effort for *S. mekongi* and hookworm egg counts. By contrast, the same efforts showed only a minimal increase for *O. viverrini* egg counts.

## Discussion

PZQ is the drug of choice against most trematode infections, including schistosomiasis and opisthorchiasis. To our knowledge, PZQ dose comparison studies have not been described for *S. mekongi*. Dose comparison studies for *O. viverrini* have been conducted, but most studies relied on an insensitive diagnostic approach, i.e., single stool specimen examination before and after drug administration. The accuracy of diagnosis, which is particularly important for estimating cure rates, can be improved by examining multiple Kato-Katz thick smears derived from a single or multiple stool specimens [30].

In this study, *S. mekongi* cure rate after administration of 75 mg/kg PZQ (80.8%) was not significantly higher than the cure rate obtained after a single dose of 40 mg/kg (75.0%) when assessed with the maximum sampling effort of 9 Kato-Katz thick smears. The cure rate from either regimen was largely overestimated if diagnosis was based on a single Kato-Katz thick smear. Studies based on fewer Kato-Katz thick smears are more likely to overestimate cure rate and be less diagnostically sensitive to detect any differences in dose comparisons. Two small studies carried out in the 1980s on *S. mekongi* infection reported high cure rates with 60 mg/kg PZQ (90.9% and 97.5%, respectively) [5], [6] when analyzing 2–3 stool specimens but using different stool diagnostic techniques (Kato-Katz+modified Ritchie's and Stoll's, respectively). Similarly in a recent multi-country randomized trial comparing single 40 mg/kg and 60 mg/kg PZQ in children aged 10–19 years, with infections diagnosed by two stool specimens (duplicate Kato-Katz thick smears per specimen), the 21-day posttreatment follow-up was reported as 92.8% with 60 mg/kg, which was not a significant improvement against *S. mansoni*, *S. haematobium*, or *S. japonicum* infections compared to the standard 40 mg/kg [31]. Consistent with results obtained from this recent trial, our study did not document a significantly improved cure rate (days 28–30 posttreatment) with an even higher total dose (75 mg/kg dose) for *S. mekongi*, even with higher diagnostic sensitivity from greater stool sampling efforts. However our additional sampling effort did observe a cure rate for 40 mg/kg about 15% lower than rates reported in the multicenter trial.

*O. viverrini* cure rate after administration of 75 mg/kg PZQ (96.6%) was significantly higher than the cure rate obtained after a single dose of 40 mg/kg (71.4%) when assessed with the maximum sampling effort. However, if the cure rate had been based on results of single Kato-Katz thick smear before and after drug administration, as often the case in community-based surveys, no significant difference would have been found. Cure rate was particularly overestimated when based on a single Kato-Katz thick smear in this study for a 40 mg/kg dose (94.3%), similar to high, and most likely overestimated cure rates (91–100%) reported from previous studies using the same dosage and only a single stool examination [9], [10], [12]. Cure rates which were reported as 100% after administration of 75 mg/kg PZQ (divided into three doses) were also likely overestimated in previous studies [7], [13].

Our study therefore provides supportive evidence that a 75 mg/kg total dose of PZQ is highly efficacious against *O. viverrini* and *S. mekongi* infections in school-aged children from Lao PDR. The total dose was divided into two doses instead of three and had a 24-hour profile of common adverse events similar to a single 40 mg/kg dose. Two doses, instead of three, are operationally and logistically more feasible, but clearly single-dose regimens are the preferred option for large-scale preventive chemotherapy programs. The small size of our study, however, limits detecting a difference in the nature or frequency of adverse events between the two regimens.

The non-significant difference between the two doses to cure *S. mekongi* infections should be interpreted with caution. Again, this may result from the study's small sample size and it would therefore be valuable to investigate a larger sample. In addition, most of the children included in our study only had low intensity infections while cure rate achieved by PZQ has been shown to be influenced by the infection burden [32]. Some authors have argued that egg reduction rate is a more appropriate indicator than cure rate for drug efficacy evaluation [33], [34]. We assessed both cure and egg reduction rates. Importantly, we found very high egg reduction rates (>99%) against *O. viverrini* for both treatment regimens regardless of the sampling effort. For *S. mekongi*, considering 9 Kato-Katz thick smears as the diagnostic ‘gold’ standard, a somewhat lower egg reduction rate was observed with a single 40 mg/kg dose of PZQ compared to the higher split dose (96.4% *vs.* 98.1%). At the lower sampling effort, higher egg reduction rates were observed (97.9% and 99.6%, respectively). These data suggest that the worm burden sharply declined from either dose regimen, which was found using either minimal or maximal diagnostic effort. This may be explained by the low posttreatment infection intensity of the non-cured children given either dose. The geometric mean egg counts in the two PZQ regimens were very similar. The public health goal of preventive chemotherapy is to reduce morbidity, which is indirectly assessed using egg reduction rates. Our results suggest that PZQ, given at a single oral dose of 40 mg/kg, is suitable to achieve this goal, particularly against *O. viverrini*.

At baseline, the relative increase of sensitivity by multiple sampling was relatively low, especially for *O. viverrini* and hookworm infections. By contrast, multiple sampling was important after treatment, when infection prevalence and intensity were much lower. As a result, the sensitivity of the first Kato-Katz thick smear was much lower after treatment than at baseline, with a 4-fold lower and 3-fold lower sensitivity to detect *O. viverrini* and *S. mekongi* infections, respectively.

A single Kato-Katz thick smear is known to have a low sensitivity for the diagnosis of *O. viverrini*, especially for low intensity infections [20]. For *S. mekongi*, the low sensitivity of a single Kato-Katz thick smear to detect this fluke observed in the present study agrees with previous findings obtained from investigations focusing on *S. mansoni* and *S. japonicum* [18], [19], [22], [35], [36]. Studies on the sensitivity of the Kato-Katz technique for diagnosis of *S. mekongi* are generally lacking.

For *O. viverrini* and hookworm diagnosis, the sensitivity of a single Kato-Katz thick smear to detect infection at baseline was fairly high. For hookworm, this was in contrast to previous studies from Côte d'Ivoire [37], [38], Ethiopia [18], and Tanzania [39], where the sensitivity of a single Kato-Katz thick smear varied from 18% to 53%. However, after drug administration, when the overall *O. viverrini* infection intensity of our cohort of children became low (<10 EPG), this study indicates the need for multiple Kato-Katz thick smear examinations, ideally performed on stool specimens collected over consecutive days for a more accurate estimation of the cure rate.

Helminth eggs are non-randomly distributed within a stool specimen because the intestinal content is not uniformly mixed [40] and may affect the sensitivity of detecting an infection and fecal egg count estimates from a single Kato-Katz thick smear. Important day-to-day variation in egg output has been thoroughly documented for *S. mansoni* and *S. japonicum* [19], [21], [22], [35]. By contrast, *O. viverrini* egg output was found to be relatively consistent over a period of several days in hospitalized patients [41]. Of note, *Schistosoma* egg shedding dynamics are additionally affected by retention of eggs in intestinal and liver tissues and the lower fecundity of female worms.

We have compared the relative importance of intra-specimen and day-to-day variation of fecal egg counts before and after PZQ administration and determined its effect on evaluating anthelmintic drug efficacy. Previous research has shown that the examination of fewer specimens from different days proved to be superior than examining multiple Kato-Katz thick smears from a single stool specimen for more accurate estimates of the ‘true’ infection status for *S. mansoni* [19], [22]. In the present study for *S. mekongi* and hookworm infections, examination of one Kato-Katz thick smear per stool specimen, with specimens collected over a 3-day period (3×1 sampling scheme), resulted in higher prevalence and mean infection intensity than three Kato-Katz thick smears taken from the first stool specimen (1×3). For *O. viverrini*, however, the 3×1 and 1×3 sampling scheme revealed the same prevalence estimates. Since repeating the collection of a stool specimen over consecutive days is more costly, logistically more cumbersome, and negatively impacts on study compliance, examination of multiple Kato-Katz thick smears from a single stool specimen should be considered as a suitable approach for community surveys of helminth infections. Similar observations have been made before for the diagnosis of *Clonorchis sinensis* [42].

*S. mekongi* is known to be endemic in certain areas of the Mekong River basin [25], [43]–[45], while *O. viverrini* and hookworm species are widely distributed across Lao PDR [46]–[48]. Point prevalences as high as those observed in the present study for *S. mekongi* (87.8%), *O. viverrini* (98.9%), and hookworm (96.7%), based on a rigorous diagnostic effort, have rarely been described in the literature. Yet, our findings corroborate with a recent risk profiling study in more than 50 villages of Champasack province, where *O. viverrini* prevalences were above 80% in most villages, with particularly high prevalences observed in villages in close proximity to the Mekong River [24]. WHO surveyed selected villages on Khong Island (an island also situated along the Mekong River, only 10 km from our study site) prior to starting schistosomiasis control campaigns in the late 1980s, and found a similarly high *S. mekongi* prevalence (87.8%) as reported here [49].

Studies carried out in rural provinces of southern Lao PDR (Champasack and Saravane) reported prevalences of *O. viverrini* and hookworm ranging from 18.8% to 70.8% and from 12.5% to 46.1%, respectively [46], [47], [50], [51]. Infection prevalence is known to vary locally [46], which may partially explain the difference between prior estimates and those found in this study. However, previous prevalence estimates were based on a single Kato-Katz thick smear, while 9 Kato-Katz thick smears were examined in the present study. *O. viverrini* infection prevalence probably includes MIF infections since co-infections are common, and polymerase chain reaction (PCR) techniques on stool specimens taken from the same study area in southern Lao PDR [52] have demonstrated that MIF eggs cannot easily be distinguished microscopically from *O. viverrini* by the Kato-Katz technique [26].

In conclusion, the present study found that the added benefit of multiple Kato-Katz thick smear examination and repeated stool sampling depends on the helminth species and baseline infection intensity. Thus, in the present setting in Lao PDR, where *O. viverrini, S. mekongi*, and hookworm are all highly endemic, estimating the baseline prevalence and intensity of infection for these species with a single Kato-Katz examination may be acceptable. By contrast, estimating the prevalence of infection after treatment by the Kato-Katz technique requires multiple thick smears, ideally taken from multiple stool specimens because the positive predictive value is lower (both lower prevalence and lower geometric mean fecal egg count after treatment). A single Kato-Katz thick smear after treatment will considerably overestimate cure rate, but only minimally influences egg reduction rates. A rigorous diagnosis approach is necessary for estimating ‘true’ cure rates, as it has been previously demonstrated in studies on *S. mansoni* [30], [53]. For anthelmintic drug evaluations with emphasis on egg reduction rates, a single Kato-Katz thick smear before and after treatment might suffice. In our view, multiple stool examination should nonetheless be considered in a subsample of the population surveyed in order to improve the monitoring of large-scale control programs, provide reasonable estimates on infection prevalence and intensity, and detect subtle changes in drug efficacies that might indicate the emergence of drug resistance development.

## Supporting Information

Checklist S1 **CONSORT Checklist.**
(PDF)

Protocol S1 **Trial Protocol.**
(PDF)
