# Supplementary material for: Efficacy of Praziquantel against Schistosoma mekongi and Opisthorchis viverrini: A Randomized, Single-Blinded Dose-Comparison Trial
Source: PLoS Negl Trop Dis. 2012 Jul 24;6(7):e1726. doi: 10.1371/journal.pntd.0001726 (PMC3404075; doi:10.1371/journal.pntd.0001726)
Supplement: Protocol S1 — Trial Protocol. (PDF) [file pntd.0001726.s002.pdf]

PROTOCOL: "Praziquantel dose comparison in Lao schoolchildren"

STUDY PERIOD: February - March 2007; May 2007 for 90-day follow up
INVESTIGATORS:

1. Field Investigators: Dr. med. T.K. Mak, L. Lovis, Dr. med. P. Soukhathammavong, Dr. med. K. Phongluxa, Dr. med. Y. Vongh
2. Scientists: Ass. Prof. Dr. med. K. Akkhavong, Prof. Dr. med. C.F. Hatz, Dr. J. Keiser, Dr. P. Odermatt, Dr. med. O. Rasphone, Prof. Dr. M. Tanner, Ass. Prof. Dr. J. Utzinger, Dr. P. Vounatsou

I. INTRODUCTION

Praziquantel is accepted as the current first choice for safe, effective treatment for human trematode infections. Deworming programmes targeted towards school-age children against schistosomiasis are based on reducing the intensity of infection with the aim of reducing the associated burden of disease. The World Health Organization recommendation on standard dose based on body weight for deworming programmes is a single dose of oral praziquantel between 40 and 60 mg/kg.[1] Praziquantel doses to treat *Opisthorchis viverrini* infection are commonly based on a total dose of 75 mg/kg in divided doses.

In Lao PDR, praziquantel is recommended for several parasitic infections but in different doses depending on the type of infection[2]:

1. Schistosomiasis: 60 mg/kg into 2 doses or 40 mg/kg single dose mass treatment
2. Cysticercosis: 75 mg/kg/day divided into 3 doses x 15 days
3. Paragonimiasis: 75 mg/kg/day divided into 3 doses x 2 days
4. Opisthorchiasis: 75 mg/kg divided into 3 doses x 1 day or 40 mg/kg single dose mass treatment

Although praziquantel is known to be effective against all five of the schistosome species causing disease in humans, there are only 2 small published clinical trials on praziquantel cure rates against *Schistosoma mekongi*. The first was a small nonrandom crossover study of 11 infected Laotian immigrants within one family, who received either praziquantel 60 mg/kg in 3 divided doses with food every 4 hours or a placebo, then the opposite treatment 2.5 months later.[3] Stool samples were examined just 2.5 weeks following treatment using the Kato or Ritchie technique and 10/11 of subjects were considered cured. The second was a study of 84 Cambodian refugees who received praziquantel at 60 mg/kg in 2 divided doses 6 hours apart and admitted to hospital to observe side effects for 24 hours.[4] Stools were examined with 2 aliquots per sample of 3 separate samples using the Stoll technique. The mean egg count prior at baseline was just 61.6 eggs per gram. Side effects were nonsevere and transient. Follow up at 1 month found 77/79 egg-free and at 2-3 months 72/72 were egg-free.

These two studies involved infected individuals relocated to nonendemic areas using 60 mg/kg total doses. A controlled trial using 40 mg/kg or a comparison between two praziquantel doses for superiority against *S. mekongi* has not been described. Furthermore, optimising the Praziquantel dose and interval for treating schoolchildren co-infected with *S. mekongi* and *O. viverrini* has not been studied.

II. OBJECTIVE

The objective is to conduct a dose comparison study to test field effectiveness of two different doses and schedules of oral Praziquantel in a population of Lao schoolchildren in a *S. mekongi* and *O. viverrini* endemic area. The primary outcome of interest is cure rate of trematode infections (primary interest is *S. mekongi* and *O. viverrini*). This is a dose comparison study of two established ranges of Praziquantel: 75 mg/kg and 40 mg/kg (the standard mass campaign single dose of Praziquantel).

III. HYPOTHESES

1. Praziquantel 75 mg/kg, divided into 2 doses (50 mg/kg + 25 mg/kg 4 hours apart) and taken with a carbohydrate snack, has a cure rate (π) of *S. mekongi* infection significantly superior to the standard mass treatment dose of 40 mg/kg single dose.

   Null hypothesis: $H_0 = \pi_{TREATMENT} - \pi_{REFERENCE} \leq \delta$
   where δ is the minimum difference of clinical significance. For H_0, δ=0 for superiority trial

2. Praziquantel 75 mg/kg treatment divided into two doses (2/3 at first dose, 1/3 at second dose) has an equally acceptable side effect profile as the standard active comparator.

III. Study design: Dose comparison study that is randomized, single-blinded, and 1:1 allocation in one facility.

2. Dose A: Praziquantel 75 mg/kg (50 mg/kg + 25 mg/kg, 4 hours apart)

3. Dose B: Praziquantel 40 mg/kg PO one dose

4. Primary outcomes of Interest

1. Proportion cured of *S. mekongi* and *O. viverrini* infection with Praziquantel 40 mg/kg at Day 30 and Day 90.
2. Test for significant difference in cure rate with PZQ 40 vs 50+25 at Day 30 & Day 90.
3. Test for significant difference in quantitatively reduced intensity of *S. mekongi* and *O. viverrini* infections (incomplete cure) between Praziquantel 40 and 50+25 at Day 30 and Day 90.
4. Side effect profiles comparing the two doses and those infected vs uninfected at baseline

5. Secondary outcomes of Interest

1. Test for significant difference in intensity of infections of soil-transmitted helminths.

6. Study population & site

The entire population of children attending the single school located on Long Island, Champasak Province, Laos will be invited to participate following informed and signed consent from parents or guardians. This school has a reported 309 students (151 aged 6-10 yrs, 158 aged 11-16 yrs). Our 2006 cross-sectional study on this island showed 69-73% *S. mekongi* and 77-89% *O. viverrini* infections in these age groups as well as soil transmitted helminth infections, therefore an estimated 210 schoolchildren are *S. mekongi* positive. Each of the 4 villages on this island has approximately 500 villagers of Lao ethnicity with rice/tobacco farming as the main livelihoods.

7. Timing of Study: Baseline Day 0; Proportion cured at Day 30 & Day 90

IV. EFFECTIVENESS ASSESSMENTS

1. Clinical measurements

1. Baseline Schoolchildren morbidity questionnaire – all subjects
2. Baseline Height, Weight, MUAC – all subjects
3. Side effect – medical review of subjects with complaints 3 hrs after first dose (T_{max})
4. Full side effect questionnaire administered to all subjects 24 hours following PZQ

2. Laboratory measurements

1. Kato-Katz (K-K) thick stool smears determine infection status. Minimum acceptable number is one K-K slide for every stool specimen x 3 specimens (K-K x 3)
2. Detailed K-K examination of intra-variation within one stool sample collected at 1) baseline and 2) 30 day follow up provides high quality of baseline infection status and 30-day follow up (n=120, 60 in each arm) nesting Protocol III K-K 3 x 3 study by Leonore (MSc thesis)
3. 10% random selection of K-K slides will have a second reading as quality control by the senior parasitologist.
4. Hemoglobin at baseline and 90-day follow up; daily control check of Hemocue machine
5. SAF sample using first specimen of stool in SAF preservative x 1

4. Randomization and Allocation concealment

.

1. Following line listings of the school attendees with measured weights and informed, signed consent of participants and parents, a random numbers table will be used to assign the two groups randomly.
2. The treatment doctor is not the same doctor who records the side-effects, blinded to the higher dose group.
3. Doses are based on weight rounded to the nearest 150 mg. The Praziquantel tablet (Distocide, Korean manufacturer) is a white tablet shaped as a rounded rectangle and pressed with 3 scorelines. Each tablet is 600 mg, and each quarter tablet is 150 mg.
4. The doses are prepared in advance by two study leaders not involved in administrating the treatment. Prior to treatment day, all doses are prepared using small envelopes that are labeled with the dose number, study unique identification number, the child's name, and weight using the line listing. After the dose envelopes are prepared the Group Allocation list will be sealed in an envelope.
5. On treatment day, the child is asked to stand again on the weigh scale. The weight is retaken as a control to ensure the weight corresponds to the written weight on the dose envelope. This serves as a second assurance the dose in the envelope was calculated using a correct weight. The doctor administering the treatment was not involved in the randomization or preparing doses in envelopes.
6. Each dose is given with 2 soupspoons of sticky rice and bottled water.
7. Single-blind
   a. the treatment doctor is not the same doctor who records the side-effects.
   b. The Kato-Katz microscopists are blinded to those who were Dose A or B subjects.
   c. The ultrasonographer at 90-day follow up is blinded to those which were Dose A or B subjects.

V. FIELD PROCEDURES

1. Treatment of subjects (see Figure 1)

1. Inclusion: all schoolchildren attending Don Long
2. Exclusion: acutely ill children; at clinical discretion and following review of the morbidity questionnaire.
3. Record the number of absent children and reason to Day 90
4. Record the number of withdrawn subjects and reason to Day 90
5. Information and written consent from parents or guardians of every schoolchild
6. Baseline
   a. Short morbidity questionnaire
   b. Ht, Wt, MUAC, Hemoglobin
   c. Hepatobiliar & splenic ultrasound
   d. Kato-Katz 3 x 3 for infection status
   e. SAF x 1 of first sample
   f. Frozen stool x 1 of first sample
7. Randomization of subjects (both infected and noninfected) into 2 equal arms
8. (Group A) Praziquantel 75 mg/kg total dose or (Group B) 40 mg/kg

9. Each dose taken PO with 2 soupspoons of sticky rice to increase bioavailability
10. Compliance: Treatment is directly observed.
11. Reported side effect questionnaire at 3 hrs
12. Elicited side effect questionnaire at 24 hours
13. Repeat baseline measures (6a to e) at:
    a. Day30 (except hemoglobin)
    b. Day 90 (including hemoglobin)
14. Universal treatment with Albendazole 400 mg PO one dose when study completed after Day 90 follow up.

3. Logistics (see Figure 1)

All studies are integrated and take place on Don Long in Feb and March 2007.
Protocol I: "Follow up of morbidity in Khong cohort" (Tippi)
Protocol II: "Praziquantel 40 vs 60 in Lao schoolchildren" (Tippi)
Protocol III: "Day-to-day and intra-specimen variation" (Leonore)
Protocol IV: "Cure rate at Day 14/30/90"(Leonore) nested in Protocol II; new interval Day 14

VI. STATISTICS

1. Unit of randomization = individual

Simple randomization of treatment (40 mg/kg single dose vs. 50+25 mg/kg) within each class to avoid cluster sampling (eg. all in same class receive same treatment) that would require a higher number of subjects. This is slightly more complicated on the Study Treatment day 0 but thereafter both subject groups are treated the same.

2. Sample size calculation

1. Superiority hypothesis; 1-tailed test
2. Add + 15% for dropouts

π_R = estimated proportion cured of reference dose 40 mg/kg = **0.70**
π_T = estimated proportion cured of treatment dose 50/mg kg + 25 mg/kg = **0.80, 0.85, or 0.90**

Sample size estimate based on Formula (7) in

**Minimum treatment effect size $\pi_T - \pi_R$**	**Number of subjects per group**					
	**Type I error: alpha = 5%**			**Type I error: alpha = 10%**		
	**Power 0.8**	**Power 0.7**	**Power 0.6**	**Power 0.8**	**Power 0.7**	**Power 0.6**
10%	228	174	134	167	121	88
15%	92	70	55	68	49	36
20%	47	36	28	35	25	18

The sample size formula used in these calculations is based on the asymptotic normal method (formula 7) in Sahai & Khurshid.[5]

If there are 300 schoolchildren and estimated 70% (210) are positive on stool for S. mekongi infection, this study would be 80% powered to detect a 15% clinical difference (type I error 5 %) between the different dose regimens.

3. Analysis

Primary analysis is by Modified Intention-to-Treat (analysed based on initial treatment assignment of subjects who received at least 1 dose, but ignoring protocol violation (eg. missed second dose), crossover and withdrawal). Secondary analysis will be Per Protocol. Results will compare confidence intervals with the prespecified sensitivity to detect a superiority of cure rate ≥ 15%.

ETHICAL CONSIDERATIONS for “Praziquantel 40 vs 50+25 in Lao schoolchildren”

This study is a single-blinded dose comparison study in schoolchildren using two standard ranges of Praziquantel.

This study is submitted to the Lao National Ethics Committee for Health Research (NECHR) for approval as a subprotocol to the present NECHR-approved research project “Food-borne trematodiasis: Role in hepatobiliar and intestinal morbidity and risk patterns for infection in ecological and socio-economic distinct settings of Southeast Asia” which received NECHR clearance on 1 March 2005. Following Lao NECHR review, their response and a copy of this protocol will then be submitted to EKBB (Switzerland).

Subjects: Schoolchildren aged 5 to 16 years are the subjects of this study and they will be informed and given clearly the opportunity to withdraw at any time. Informed signed consent from the parent or guardian will be obtained for every participant and clearly advised two different doses of the same drug are standard ranges used to treat worm infections; both doses are effective and those in the lower dose group receive the standard mass campaign treatment.

Dose & side effects: Both doses of Praziquantel as a single 40 mg/kg dose or total of 75 mg/kg dose are accepted within Lao Ministry of Health published guidelines. The dose regiment of 50 mg/kg + 25 mg/kg, given four hours apart is not a standard schedule however both 50 mg/kg and 25 mg/kg dosages are used in Lao guidelines. The first dose of 50 mg/kg is within the recommended level of 40 to 60 mg/kg as a single dose for schistosomiasis. The interval between dosing is standard at 4 hours and the second dose is a standard approved amount of 25 mg/kg when used for divided doses. Possibly higher number and grade of side effects may occur in the higher dose group. Side effects will be closely monitored by medical staff from this study team and any severe or unusual outcomes will be reported.

References

1. Montresor A, Crompton D, Gyorkos TW, Savioli L. Helminth control in school-age children. A guide for managers of control programmes. *World Health Organization, Geneva* 2002.
2. Ministry of Health Lao PDR. Diagnosis & treatment in district hospitals, 2nd ed. 2004.
3. Nash TE, Hofstetter M, Cheever AW, Ottesen EA. Treatment of Schistosoma mekongi with praziquantel: a double-blind study. *Am J Trop Med Hyg* 1982;31(5):977-82.
4. Keittivuti B, Keittivuti A, O'Rourke T, D'Agnes T. Treatment of Schistosoma mekongi with praziquantel in Cambodian refugees in holding centres in Prachinburi Province, Thailand. *Trans R Soc Trop Med Hyg* 1984;78(4):477-9.
5. Sahai H, Khurshid A. Formulae and tables for the determination of sample sizes and power in clinical trials for testing differences in proportions for the two-sample design: a review. *Stat Med* 1996;15(1):1-21.
6. The European Agency for the Evaluation of Medicinal Products Evaluation of Medicines for Human Use (EMEA) J. Points to consider on switching between superiority and non-inferiority. CPMP/EWP/482/99. 27 July. 2000.
